# Resisting Potato Cyst Nematodes With Resistance

**DOI:** 10.3389/fpls.2021.661194

**Published:** 2021-03-25

**Authors:** Ulrike Gartner, Ingo Hein, Lynn H. Brown, Xinwei Chen, Sophie Mantelin, Sanjeev K. Sharma, Louise-Marie Dandurand, Joseph C. Kuhl, John T. Jones, Glenn J. Bryan, Vivian C. Blok

**Affiliations:** ^1^Cell and Molecular Sciences, The James Hutton Institute, Dundee, United Kingdom; ^2^School of Biology, University of St Andrews, St Andrews, United Kingdom; ^3^School of Life Sciences, University of Dundee, Dundee, United Kingdom; ^4^INRAE UMR Institut Sophia Agrobiotech, Sophia Antipolis, France; ^5^Entomology, Plant Pathology and Nematology Department, University of Idaho, Moscow, ID, United States; ^6^Department of Plant Sciences, University of Idaho, Moscow, ID, United States

**Keywords:** nematodes, *Globodera*, resistance, virulence, molecular markers, genomics, potato breeding, integrated pest management

## Abstract

Potato cyst nematodes (PCN) are economically important pests with a worldwide distribution in all temperate regions where potatoes are grown. Because above ground symptoms are non-specific, and detection of cysts in the soil is determined by the intensity of sampling, infestations are frequently spread before they are recognised. PCN cysts are resilient and persistent; their cargo of eggs can remain viable for over two decades, and thus once introduced PCN are very difficult to eradicate. Various control methods have been proposed, with resistant varieties being a key environmentally friendly and effective component of an integrated management programme. Wild and landrace relatives of cultivated potato have provided a source of PCN resistance genes that have been used in breeding programmes with varying levels of success. Producing a PCN resistant variety requires concerted effort over many years before it reaches what can be the biggest hurdle—commercial acceptance. Recent advances in potato genomics have provided tools to rapidly map resistance genes and to develop molecular markers to aid selection during breeding. This review will focus on the translation of these opportunities into durably PCN resistant varieties.

## Introduction

Potato is now the third most important food crop after rice and wheat for human consumption (CIP, 2020). Being an important source of carbohydrates, potato provides more protein and minerals than any other staple crop (Birch et al., 2012). With the increasing demand from a growing world population, optimised pest and disease management are of increasing importance for the sustainability of this crop. Potato cyst nematodes (PCN) are the most economically important parasitic nematodes of potato. They are soil inhabiting biotrophic sedentary endoparasites (Wyss, 1997) and are specialised pathogens of solanaceous species (Sabeh et al., 2019). The global economic impact of PCN has not been fully quantified, however, even a small infestation can incur considerable costs, including loss of income to growers, closure of local and international markets as well as expenses for regulatory activities such as surveys and monitoring systems (Hodda and Cook, 2009). There are estimates that the two species, *Globodera rostochiensis* and *G. pallida*, are responsible for the loss of 9% of the crop worldwide (Turner and Subbotin, 2013). A potential third PCN species was reported by Skantar et al. (2011) and has subsequently been described as *G. ellingtonae* (Handoo et al., 2012). It has been detected in Oregon and Idaho in the United States, and in the Andean region of South America (Lax et al., 2014). However, its pathogenicity to potato is inconsistent (Zasada et al., 2019).

PCN co-evolved with their hosts from the genus *Solanum* in South America (Canto Saenz and Mayer de Scurrah, 1977; Stone, 1985). For *G. pallida*, this co-evolution is estimated to have taken place over 20 million years, with a northward expansion from the south of Peru around Lake Titicaca (Picard et al., 2004, 2007). Both nematode species were probably introduced and became established in Europe in the 1850s as a consequence of the Irish potato famine (Evans et al., 1975), when tubers, with contaminated soil attached, were collected and brought to Europe as breeding material for late blight resistant potatoes. In the 1880s, damage to potato crops from nematodes was noted in Germany, and from the early 1900s in the United Kingdom (Evans et al., 1975). PCN have subsequently been spread, mainly via contaminated seed potatoes, to almost all countries where potato is grown. To date, they have been detected in 126 countries (79 for *G. rostochiensis* and 55 for *G. pallida*) (CABI, 2020a,b). PCN are quarantine pathogens, which means that phytosanitary regulations are applied to prevent the introduction or spread within a country, and once detected, eradication or containment measures are applied (reviewed in Pickup et al., 2018). Protection of seed land from infestations is vital to prevent spread into non-infested land within the EU, and testing of seed land prior to planting is required by the EU Council Directive 2007/33/EC. The regulations that apply to PCN provide a framework for the potato industry, but despite these regulations PCN continues to spread. The long delay between introduction and detection, which may require several crop cycles, confounds interpretation of how effective these regulations may be. The widespread presence of these nematodes in East Africa provides a contemporary example of the failure to prevent spread and the devastating consequences incurred by subsistence farmers who have become dependent on potato for their livelihoods (Mburu et al., 2020). Both species of PCN are also widespread in Algeria, the largest potato producer in Africa (Djebroune et al., 2021).

**FIGURE 1:**
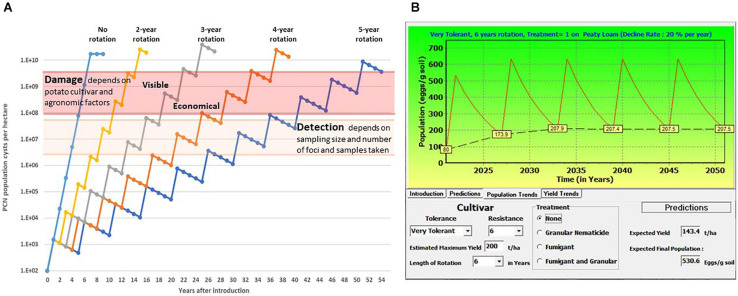
**(A)** Schematic illustration of the increase of potato cyst nematodes (PCN) cysts with different rotation intervals, and the time until detection and damage thresholds are reached. This simplified sketch shows a natural decrease of viable cysts of 25% per year, and a 15-fold multiplication rate per year, no other factors are considered. **(B)** This illustrates the predictions of a model in which different factors including initial population level (in this example 80 eggs/g soil) soil type, nematicide treatment and rotation length, and cultivar characters including tolerance and resistance, can be altered to predict population and yield trends using a specified decline rate of 20% (https://pcncalculator.ahdb.org.uk).

Disease symptoms caused by PCN are initially minimal, due to low infection levels in localised patches. Such symptoms are frequently interpreted as the effects of abiotic stress, and infestations frequently remain unidentified for many years until reduced yields or premature plant senescence are observed. PCN population dynamics has been studied since the 1950s, in order to predict yield losses by modelling different parameters such as level of field infestation at the start of the season, use of nematicides, soil type, rotation length and use of resistant/susceptible potato cultivars (e.g., Seinhorst, 1982; Been and Schomaker, 1998; Trudgill et al., 2014). Online tools such as NemaDecide^1^ and PCN Calculator^2^ were developed in an attempt to allow potato growers to simulate expected tuber yields and PCN population development when different conditions are applied. Figure 1A shows the time that passes between introduction of PCN to a field and reaching the detection/damage thresholds under different rotation schedules of susceptible crops in a very simplified model, only considering a constant multiplication rate, set at 15-fold per annum, and a fixed rate of natural decline, set at 25% per annum. In Figure 1B, an example of the predictions for population levels and yield trends is shown. The parameters used such as initial population level, decline rate, soil type, nematicide treatment, rotation length, cultivar resistance and tolerance can be adjusted with the PCN Calculator for *G. pallida*^2^.

The fact that PCN cysts are highly durable, and viable eggs can survive for decades in the absence of a host (Turner, 1996), together with difficulty in detection of new infestations, makes preventing the spread of PCN very challenging. An example of a successful eradication is from Western Australia, where in the late 1980s a total area of 35 ha was found to contain *G. rostochiensis.* This area was declared PCN free in 2010. However, at least part of this land has been converted to housing estates, precluding further sampling (Collins et al., 2010; IPPC, 2010). In Idaho, a containment and eradication programme is in progress following the detection in 2006 of two potato fields infested with *G. pallida* (Skantar et al., 2007; USDA, 2007). Preventing spread and eradication with soil fumigants is the focus of the regulatory programme in Idaho^3^. When PCN is well established but not widespread, preventing spread to uninfected fields becomes paramount. With widespread infestations, management involves various strategies (Back et al., 2018), including crop rotation, chemical and biological control, bio-fumigation and trap cropping. This review focuses on the use of host resistance to manage PCN.

## Host Nematode Interactions

Potato cyst nematodes have long lasting biotrophic interactions with their hosts. Cysts, located in the soil after detaching from host roots, contain several hundred eggs, each of which contains a juvenile nematode (J). The moult from J1 to J2 takes place within the egg once embryogenesis is complete. The unhatched J2 is protected by a chitinous eggshell which is an impermeable barrier and provides physical protection against potential bacterial or fungal pathogens. Furthermore, the unhatched J2 is dehydrated and suspended in a perivitelline fluid that contains a high concentration of trehalose, which provides protection against low winter temperatures (Perry, 2002). Hatching of PCN eggs is triggered by exposure to chemicals released from the host roots, termed host root hatching factors. The requirement for hatching factors in root exudates to stimulate hatching of the J2s provides a means for synchronisation of the PCN life cycle with the presence of a host plant (Masler and Perry, 2018). The presence of hatching factors triggers a calcium dependent change in eggshell permeability, allowing water to enter the egg, reactivating the metabolic activity of the unhatched J2 (Clarke and Perry, 1985). The J2 nematode subsequently cuts its way out of the eggshell using its stylet (Doncaster and Seymour, 1973). Host resistance is not associated with the ability to reduce hatch of PCN—root exudates of resistant plants induce similar levels of hatch as those from susceptible plants (Turner and Stone, 1981). After hatching, the J2 moves through the soil, locating, and invading the host root. Physiologically active roots exude an array of molecules that form chemical gradients in soil that are perceived by the nematodes, allowing their orientation and migration toward the host. Despite the importance of the chemical signalling that governs the nematode chemotaxis, advances in nematode chemical ecology are relatively recent (Torto et al., 2018). To date, there is no evidence that host resistance affects PCN chemotaxis.

**FIGURE 2:**
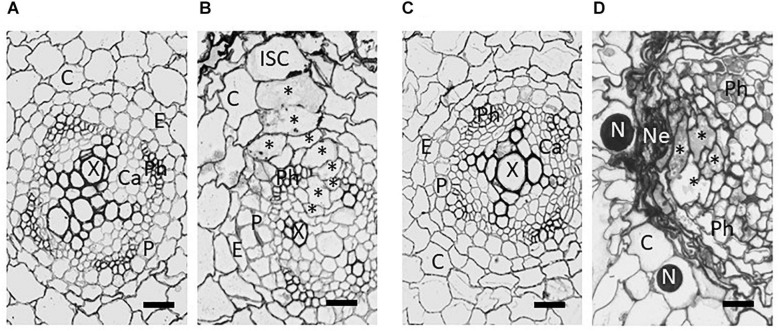
Brightfield images from tangential sections of potato roots showing development of syncytia induced by *Globodera pallida* in the roots of susceptible *Solanum tuberosum* cv. Pentland Ivory **(A,B)** and resistant reaction of Sa_12601 [*S. tuberosum* ssp. *andigena* CPC 2802 (*H3*)] **(C,D)**. **(A)** Uninfected root and **(B)** syncytium at 4 dpi of susceptible cv Pentland Ivory, **(C)** uninfected and **(D)** necrosis and collapse of syncytium at 7 dpi of Sa_12601. C, cortex; Ca, pro-/cambium; E, endodermis; ISC, initial syncytial cell; N, nematode; Ne, necrosis; P, pericycle; Ph, phloem; X, xylem. Asterisks (*) indicate selected syncytial elements. Scale bars: 20 μm (from Varypatakis et al., 2020).

After penetrating the host, the nematodes migrate destructively through the root cells until reaching the inner cortical layer. Migration is facilitated by the production of a cocktail of plant cell wall degrading enzymes and modifying proteins including cellulases (Smant et al., 1998), pectate lyases (Popeijus et al., 2000), and expansins (Qin et al., 2004). The genes encoding these proteins are likely to have been acquired as a result of horizontal gene transfer from bacteria (Kikuchi et al., 2017). After reaching the inner cortical layer, the behaviour of the J2 changes and the nematode probes cells with its stylet to select an initial syncytial cell (ISC) that does not respond adversely by producing callose (Sobczak and Golinowski, 2011). Effectors are introduced into this ISC which then undergoes a transformation into a syncytium. Within the ISC the central vacuole degrades, the cytoplasm becomes enriched in subcellular organelles, and the nucleus becomes enlarged and ameboid in shape (Golinowski et al., 1996). The syncytium develops into a multinucleate structure through controlled breakdown of the cell walls separating the ISC from its neighbours, initially at the sites of plasmodesmata, with the subsequent fusion of protoplasts of adjoining cells (Grundler et al., 1998; Figure 2). Up to 300 cells can be included in the final structure. The syncytium provides a rich food source for the developing nematode but needs to be kept alive for the duration of the nematode life cycle as the nematode is not able to induce more than one such structure. The nematode produces a feeding tube during each cycle of feeding (Eves-van den Akker et al., 2015a). The precise nature of this structure remains unknown, but it likely acts as a molecular sieve and prevents damage to the syncytium when feeding.

The interactions between PCN and its host are mediated by effectors. These proteins are secreted from the nematode into the host to manipulate its metabolism to the benefit of the nematode. PCN effectors, like those of other plant-parasitic nematodes, are produced in two subventral and one dorsal gland cell. Ultrastructural studies (Hussey and Mims, 1990), as well as transcriptome analysis (Thorpe et al., 2014) indicate that effectors important in the early stages of the host-parasite interaction (including migration) tend to be produced in the subventral gland cells, while those that are important at later stages are produced in the dorsal gland cell. In terms of the interaction between PCN and its host, effectors have been characterised that assist in the migration of the nematode through the root (e.g., Smant et al., 1998), in the induction of the syncytium and suppression of host defence responses (e.g., Mei et al., 2015).

The precise details of how the syncytium is formed remain unclear. However, it is known that nematode control of several plant hormone pathways is key to this process (Gheysen and Mitchum, 2019). Manipulation of these pathways can be achieved through effectors and plant peptide hormone mimics produced by the nematode (Ali et al., 2017). In particular, the role of auxin, which is a central regulator of plant organogenesis, has been investigated during the feeding site formation. Studies of auxin synthesis and perception mutants showed that manipulation of the auxin signalling pathway plays a critical role in the development of syncytia. For example, auxin insensitive mutants are unable to support normal syncytium development, while disruption of polar auxin transport gives rise to abnormally large syncytia (Goverse et al., 2000). In addition, the rearrangement of PIN and AUX/LAX auxin transporters in developing syncytial cells, where auxin responsive promoters are also activated, indicates that changes in local auxin concentration are important during syncytium formation (Grunewald et al., 2009).

As in other pathosystems, resistance against PCN is triggered by recognition of its effectors by *R*-genes, either directly or indirectly (Sacco et al., 2009). The effector gene *RBP-1* was identified as the *Gpa2* resistance cognate avirulence (*Avr)* factor (Sacco et al., 2009). It belongs to the SPRYSEC effector gene family (secreted SP1a and ryanodine receptor domain-containing effector), which is unique to cyst nematodes. Recently, a list of *G. pallida* candidate *Avr* genes, implicated in the interaction with plants containing the *H3* or *GpaV* resistance sources, has been proposed by Varypatakis et al. (2020). The most common candidates belong to the SPRYSEC family. Other putative *Avr* candidate genes included cellulases, glutathione synthase and an orthologue of *Heterodera glycines* GLAND3, with unknown function.

Ultrastructural studies comparing the development of the syncytium in susceptible and resistant hosts showed structural differences evident within 2 days post infection (dpi) (Rice et al., 1985; Varypatakis et al., 2020). By 4 dpi susceptible plants showed incorporation of many additional cells into the syncytium and expansion into the vascular cylinder. However, no expansion was found in the resistant host apart from some degradation of cytoplasm. By 7 dpi most syncytia were degraded and compressed in a host with the *H3* resistance derived from *S. tuberosum* ssp. *andigena* CPC 2802, and extensive necrosis of neighbouring cells was also observed (Figure 2; Varypatakis et al., 2020). Failure to incorporate cells within the vascular cylinder prevents development of a viable syncytium, which is needed by the feeding nematode to progress through moults to the adult stages. Some variation in the degree of syncytial degradation has been observed and this may account for the increased number of males observed in some hosts that are “partially resistant” and have syncytia which are compromised in their nutritional capabilities. Males do not require a sustained viable syncytium to reach maturity as they do not produce eggs, and hence a shift in the sex ratio toward males has been observed with some sources of resistance (Rice et al., 1985; Sobczak et al., 2005). Modification of the host root’s functions, caused by the initiation of the feeding sites and the establishment of the resistance response with extensive necrotic area can greatly affect plant growth and lead to crop failure at high nematode densities. The destructive root penetration during PCN invasion that occurs irrespective of the resistance, can also lead to colonisation by other root pathogens, exacerbating damage to the crop.

## Virulence, Pathotypes, and Durability of Resistance

Nematode virulence is defined as the ability to reproduce on a host plant that contains specific genes, otherwise conferring resistance. Nematodes which cannot reproduce on the same resistant plant are called avirulent (Vanderplank, 1978; Turner et al., 1983; Blok et al., 2018). Virulence traits represent a characteristic of individual nematodes. Nematode populations can consist of a mixture of virulent and avirulent individuals. This proportion can change over time, depending on environmental conditions, such as use of resistant or susceptible hosts. The reproduction rate of virulent nematodes on resistant plants is typically lower than on a fully susceptible plant. Nematodes, like other pathogens, usually conform to the so-called trade-off concept, stating that resistance-breaking populations of pathogens pay a price by losing fitness on susceptible hosts compared to avirulent populations (Stearns, 1989; Laine and Barres, 2013). Nevertheless, a recent experimental evolution study (Fournet et al., 2016) found that this might not always be the case for *G. pallida*, for which virulent populations became fitter on resistant hosts, producing bigger cysts with more juveniles that hatched earlier. However, these experiments were conducted in petri-dishes with nutrient enriched agar, and thus might not represent the behaviour in the field.

In Europe, over 40 years ago, a pathotype scheme was developed (Kort et al., 1977), in order to identify which sources of resistance were most effective against European populations of *G. rostochiensis* and *G. pallid*a. *Globodera rostochiensis* was subdivided into five pathotypes, Ro1–Ro5. Ro1 and Ro4 were differentiated by their low multiplication rate on *S. tuberosum* ssp. *andigena* CPC 1673, compared to Ro2, Ro3, and Ro5. *Solanum kurtzianum* hybrid 60.21.19 differentiated Ro2 from Ro3 and Ro5, and Ro1 from Ro4. Ro3 multiplied poorly on *S. vernei 58.16/12/4*, compared to Ro5. All *G. rostochiensis* pathotypes were unable to multiply on *S. vernei* hybrid 65.346/19. *Globodera pallida* was categorised into three European pathotypes, Pa1, Pa2, and Pa3; *S. multidissectum* hybrid P55/7 differentiated Pa1 from Pa2 and Pa3, and *S. vernei (VTn)2 62.33.3* differentiated Pa2 from Pa3.

The Kort pathotype scheme provided a panel of PCN populations representing the phenotypic spectrum in Europe and identified several sources of resistance and their specificities for use by potato breeders. For *G. rostochiensi*s, Ro1 is the dominant pathotype outside South America, and breeding efforts have focused on this pathotype. The occurrence of *G. rostochiensis* in the United Kingdom is probably the result of a single introduction (Evans et al., 1975; Bendezu et al., 1998), and resistance to *G. rostochiensis* Ro1 has proven to be remarkably durable. For *G. pallida*, the situation has been more complicated and the progress from breeding has been much slower. The differentiation of the pathotypes Pa2 and Pa3 on *S. vernei (VTn)2 62.33.3* was not found to be robust, and this has led to questions about the validity of the pathotype scheme. Trudgill (1985) demonstrated that the reproduction factor of <1 used in the Kort scheme to indicate resistance was environmentally sensitive and proposed that the Pa2 and Pa3 pathotypes were a continuum (Pa2/3) that could not be distinguished reliably. Most European populations of *G. pallida* belong to the Pa2/3 pathotype, though genetic evidence supports a distinct introduction which includes the populations Chavornay and Luffness, classified as Pa3 in the Kort scheme (Hockland et al., 2012). At least three independent introductions of *G. pallida* from South America have occurred in Europe (Plantard et al., 2008; Hockland et al., 2012). In Scotland, mixtures of these three genotypes can be present in the same field (Eves-van den Akker et al., 2015b). Since this scheme was introduced, other sources of resistance to *G. pallida* have been found that are being used in breeding programmes and resistance-breaking populations of *G. pallida* have been detected. Taken together, these facts have confounded the issue of pathotype designation.

The PCN populations outside South America represent a small portion of the total genetic diversity of these species (Plantard et al., 2008; Grenier et al., 2010). This restricted genetic variation makes it possible to consider a potato breeding approach using host resistance for their control. While the *H1* resistance to *G. rostochiensis* has shown to be highly durable, the resistance sources available for *G. pallida* are not expected to be as long-lasting due the greater genetic diversity of *G. pallida* populations. Also, in general, the resistances that have been identified for this species have not been as effective. It is possible that the combination of the more heterogenous nature of *G. pallida* populations with the more genetically complex nature of the quantitative resistances involved, will require the action of two genes for very high levels of durable resistance.

The use of resistant hosts exerts a selection pressure on nematode populations that can lead to an increase in the proportion of virulent vs. avirulent individuals (Whitehead, 1991; Hockland et al., 2012). Varypatakis et al. (2019) observed this effect when using different potato cultivars challenged with *G. pallida* populations previously grown repeatedly on potato cultivars with partial resistance to *G. pallida*. Beniers et al. (2019) also used partially resistant potato cultivars to show an increase of virulence in a population when the same source of resistance was applied over generations as have others (Turner, 1990; Phillips and Blok, 2008). Ultimately, this shift could lead to resistance-breaking PCN populations. This effect has already been reported for the resistant potato cultivar Innovator in some regions in Germany (Niere et al., 2014). Pyramiding different sources of resistance to *G. pallida* is likely to be needed to produce potato cultivars with durable, broad spectrum resistance. Indeed, Dalton et al. (2013) demonstrated a synergistic effect of increased resistance when combining two different PCN resistance loci in potato and Rigney et al. (2017) showed that this effect could be replicated with several *G. pallida* populations, though the durability of this material has not yet been assessed.

## Resistance and Tolerance to Pcn and Their Assessment

Resistance and tolerance to pests and pathogens are two different strategies that plants and animals use to cope with biotic threats. Resistance to PCN is defined as the host plant’s ability to inhibit or limit reproduction, relative to a susceptible plant (Evans and Haydock, 1990; Blok et al., 2018; Pagán and García-Arenal, 2020). In contrast, tolerance toward PCN is defined as the ability of the potato to tolerate PCN infection without a reduction in yield. Unlike resistance, tolerance does not significantly affect the nematodes’ ability to infect the host and reproduce (Starr et al., 2002; Pagán and García-Arenal, 2020), and the two traits are independent of each other (Evans and Haydock, 1990; Trudgill, 1991). When confronted with high PCN population densities, resistant cultivars that are intolerant do not perform well (in terms of yield) although they will restrict reproduction of PCN, whereas susceptible tolerant cultivars allow PCN populations levels to rise and produce adequate yields (Trudgill, 1991). Combining resistance and tolerance is now regarded as essential by potato breeders in areas where PCN is widespread.

### Resistance

The cultivated tetraploid species *S. tuberosum* ssp. *tuberosum* does not show any resistance to PCN (Rousselle-Bourgeois and Mugniery, 1995). However, related wild potato species and landraces are a genetic resource for resistance to pests such as PCN, resilience to abiotic stresses and tuber quality characters. Many of them have been systematically tested for PCN resistance (e.g., Rousselle-Bourgeois and Mugniery, 1995; Castelli et al., 2003). To date, more than 50 potato species have been identified that show resistance to *G. rostochiensis* and/or *G. pallida* for at least one pathotype, in at least one accession. Wild potato species can be found in their natural habitats from the southwestern US to Argentina, with most species being native to Mexico and the Andean Highlands (Bethke et al., 2017). Initial approaches for identifying sources of resistance to PCN in potato involved the screening of over 1,200 accessions of wild species from the Commonwealth Potato Collection and led to the first identified PCN resistance locus (*H1*) from *S. tuberosum* ssp. *andigena*, which offers nearly complete resistance to *G. rostochiensis* pathotypes Ro1 and Ro4 (Ellenby, 1952). More recently, it was reported that *G. ellingtonae* does not reproduce on potato genotypes which carry the *H1* resistance locus (Whitworth et al., 2018; Zasada et al., 2019).

Based on the “gene-for-gene” concept (Flor, 1971), quantitative resistance to PCN may be conferred by products of dominant resistance (*R*) genes. These genes recognise pathogen-produced effector proteins in infected cells, encoded by *Avr* genes, and trigger defence responses, which may include a hypersensitive reaction (Van der Biezen and Jones, 1998; Davies and Elling, 2015). The latter involves cells that are committed to cell death, depriving the nematodes of an adequate food source, and thus leading to resistance. While all known genomic loci conferring resistance to *G. rostochiensis* or *G. pallida* belong to the nucleotide-binding site and leucine-rich repeat (NB-LRR) family of R genes, it is notable that other gene classes may be important in providing resistance to PCN and other cyst nematodes. For example, the *Cf-2* gene, which confers resistance against the fungal pathogen *Cladosporium fulvum*, encodes an extracellular receptor-like protein with a LRR domain, also provides resistance against *G. rostochiensis* (Lozano-Torres et al., 2012). For both *C. fulvum* and PCN, Cf-2 is activated by pathogen attempts to interfere with the apoplastic cysteine proteinase Rcr3. In addition, resistance against the soybean cyst nematode, *H. glycines*, can be underpinned by completely different molecular mechanisms that cause a toxic response specifically targeted at the syncytium. Resistance derived from the *Rhg1* locus is based on a copy number variation of multiple genes, with three of these genes contributing to full resistance in soybean: a putative amino acid transporter, an α-SNAP [soluble NSF (N-ethylmaleimide sensitive fusion)-associated protein] and a protein containing a wound-inducible domain (Cook et al., 2012). Only the function of the α-SNAP has been elucidated to some extent. The defective resistance-type α-SNAP protein accumulates preferentially in the nematode feeding site relative to its wild type counterpart, causing cytotoxicity (Bayless et al., 2016). Similarly, the resistance conferred by *Rhg4* is mediated through a defective variant form of a serine hydroxymethyl transferase expressed in the syncytium. This enzyme is otherwise essential for cellular one-carbon metabolism, causing a failure of nutrients reaching the nematode (Liu et al., 2012). No similar mechanisms against PCN have been identified to date but, interestingly, the proteins encoded by the soybean *Rhg1* gene can function in different plant families and confers resistance to PCN in potato (Butler et al., 2019).

### Tolerance

Factors that enhance tolerance of potato to PCN are not necessarily specific to PCN. Tolerance factors may involve enhanced root growth, the activation of general plant stress responses or combinations thereof, and can provide additional tolerance to e.g., drought or other pathogens (Trudgill, 1991; Blok et al., 2018). Environmental conditions have a major impact on tuber yield (Evans and Haydock, 1990), and tolerance is therefore environmentally sensitive and difficult to reliably assess.

The damage caused by PCN depends on the initial nematode density in the soil. Intolerant (sensitive) and susceptible cultivars show damage at even low rates of infestation (10 eggs/g soil). If damage is extensive, this can lead to low reproduction of the nematodes even though the plants are susceptible. Tolerant and susceptible cultivars, planted in infested land, can lead to a very high prevalence of PCN by increasing nematode population levels, while still giving acceptable yields, which thwarts PCN management programmes. But even a tolerant plant will suffer damage at high nematode densities, mostly due to reduced uptake of nutrients (Trudgill, 1987). When PCN is well-managed and populations have a low density, intolerant resistant potatoes may be grown, however, the best way to manage PCN would be the use of potato cultivars which are highly resistant and tolerant. Resistance ensures the reduction of nematode population levels and tolerance ensures reliable crop yields, even when the initial PCN levels are high (Blok et al., 2018). Tolerance to pathogens is usually not selected for in breeding programmes, therefore this trait is often lost, unless it is linked to another commercially useful trait (Trudgill, 1991). Very few quantitative trait loci (QTLs) for tolerance to cyst nematodes have been described so far (Williams et al., 2003, 2006; Ravelombola et al., 2020).

### Assessment of Resistance

**FIGURE 3:**
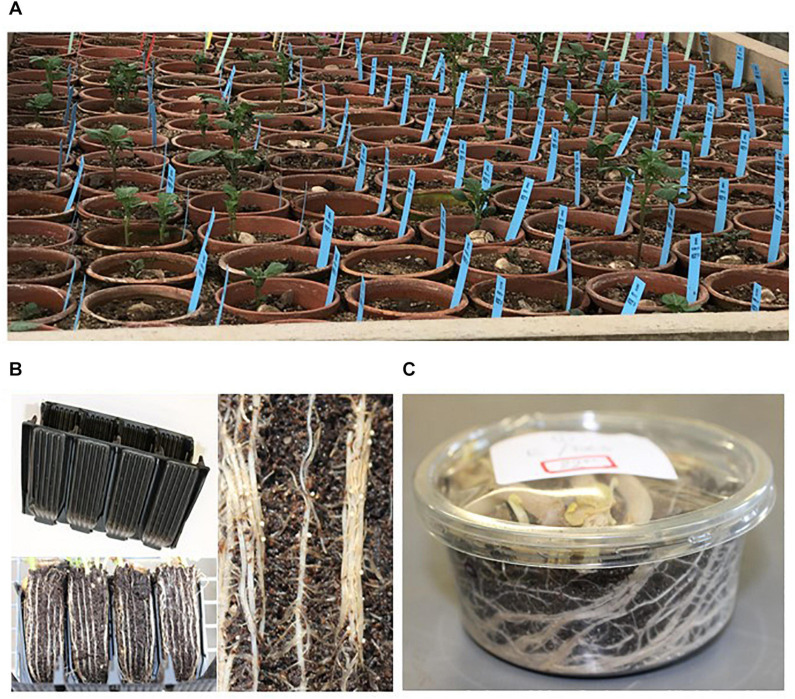
Methods for assessing PCN resistance in potatoes: **(A)** clay pots with potato plants growing in a sand/loam mixture which has been inoculated with cysts, **(B)** empty rootrainer cassette, 4 root systems in rootrainer with roots growing on surface of compost and female nematodes on root surface, **(C)** transparent canister with potato roots visible for counting female nematodes.

Any breeding programme that aims to include PCN resistance in a new cultivar requires a reliable, and ideally rapid, method for resistance screening. Screening methods to determine the level of resistance in potatoes are usually performed in a greenhouse in pots or rootrainer, or in a dark incubation chamber when using canisters (Figure 3). The choice of which test to use depends on the accuracy required, resources available and time constraints. PCN cysts, eggs or the J2 stage juveniles can be used as inoculum. For good practice, each assessment should include at least one susceptible and resistant control cultivar, be performed at least twice with a minimum of four biological replicates of each genotype grown, in a randomised planting design.

Canister tests can be used with relatively large numbers of progeny and therefore provide an efficient method to separate susceptible and resistant clones in a breeding programme. Potatoes are grown in the dark in transparent canisters with a lid, filled with moist soil and inoculated with PCN. After 8 weeks the number of female nematodes that are visible on the surface of the roots through the transparent canisters are counted (Phillips et al., 1980). The test in rootrainers is an alternative to the canister test. It is performed in a temperature-controlled greenhouse and is more labour intensive, as the plants require regular maintenance. Potato plants are grown in PCN infested soil in rootrainers for 7–8 weeks, then the number of female nematodes on the roots visible on the surface of the “root-ball” are counted after carefully removing them from the rootrainers (Strachan et al., 2019; Varypatakis et al., 2019). The most accurate assay for phenotyping potatoes is a pot test, where plants are grown for 12 weeks in a sand/loam 1:1 mixture that has been inoculated with PCN. When using cysts as inoculum, these need to be bagged to ensure that they are not counted as new cysts after the experiment is completed. The cysts are then washed out of the sand/loam mixture and counted (Fenwick, 1940; Reid et al., 2015).

The nematode reproduction rate P_*f*_/P_*i*_ (multiplication factor with P_*f*_ and P_*i*_ being the final and initial female/cyst numbers, respectively) is a measurement used to assess the relative resistance of a potato genotype. If there are very few cysts, the eggs are extracted from the cysts and counted instead to assess reproduction. The current resistance evaluation system for PCN used in Europe was introduced in 2006 (EPPO, 2006). The reproduction rate of the *G. pallida* population Chavornay on the susceptible potato cultivar Desirée is assessed, and the susceptibility is divided into 9 susceptibility groups, with 1–3 being susceptible (>100–5%), 4–6 partially resistant (<25–3%) and 7–9 resistant (<3%). This system uses an agreed set of protocols and means that resistance ratings can be applied consistently to new cultivars.

All these bioassays require specialised materials and trained staff. They are time consuming and therefore expensive. Using genetically linked molecular markers to determine the presence or absence of resistance loci is faster and allows more progeny to be screened. However, bioassays ultimately need to be performed to confirm resistance. The accuracy of molecular markers is not always absolute, as homologous recombination can separate the marker and the resistance locus in each generation, the likelihood of recombination increasing with distance between marker and resistant locus on the genetic map. This problem may be overcome when the marker resides in the resistance gene itself, or if two markers flanking the resistance are used, as two recombination events in one generation are extremely rare.

### Assessment of Tolerance

Tolerance to PCN is a complex trait and to date no molecular markers are available. Bioassays are therefore the only option to assess tolerance. To assess tolerance, the performance of different infected and non-infected potato genotypes is compared with that of genotypes with known tolerances (Cook and Evans, 1987; Trudgill, 1991). Field tests for the evaluation of tolerance are described in Trudgill (1991), whereas Arntzen and Wouters (1994) described a high correlation between pot and field evaluations of tolerance against PCN during early growth. The use of resistant and susceptible control potato cultivars with known levels of tolerance is advised in those assays, as the results are highly variable, both in replicates and in different experiments (Dale et al., 1988). If the assay is performed with and without nematicides, tolerant potatoes are expected to have the least increase of yield in conjunction with nematicide use (Trudgill and Cotes, 1983a). Tolerant cultivars often grow larger roots in heavily infested soil compared to nematicide treated or only lightly infested soil (Trudgill and Cotes, 1983b).

## Breeding for Pcn Resistance and the Development of Molecular Markers for Marker Assisted Breeding

### History of Breeding for PCN Resistant Potatoes

The modern cultivated potato derives mainly from a relatively small number of introductions into Europe from South America in the 16th, 17th, and 18th centuries centuries. By the end of the 18th century the crop had quite rapidly been adapted to tuberise in the long-day conditions found in Northern Europe through selection for early-tuberisation and higher tuber yields. However, early cultivars lacked sufficient genetic variation to provide resistance to major pests and diseases. This became a problem once the crop became widely grown as the predominant staple food crop in many countries in the 19th century. The most notable pathogens have been late blight (*Phytophthora infestans*) and latterly, PCN. This has led to the need to introgress genes for disease and pest resistance into potato varieties from the many wild and cultivated species of Central and South America, some of which can be crossed directly with cultivated potato.

Commercial potato breeding is still performed mainly at the tetraploid level, whereas many of the wild and primitive cultivated species containing disease resistance are diploid. The highly heterozygous nature of tetraploid potato breeding germplasm greatly compromises potato improvement. Potato breeding remains largely empirical and genetically unsophisticated, and most economically important traits are genetically complex, displaying continuous variation. Resistance to pest and disease threats are largely an exception, being generally controlled by the action of one or two dominant genes. This property has led to a focus on developing genetic markers for use in breeding for pest and disease resistance. Development of molecular genetic tools facilitating the mapping and cloning of genes that confer resistance has significantly accelerated the process of PCN-resistant cultivar development through marker-assisted selection (MAS) in recent years.

**FIGURE 4:**
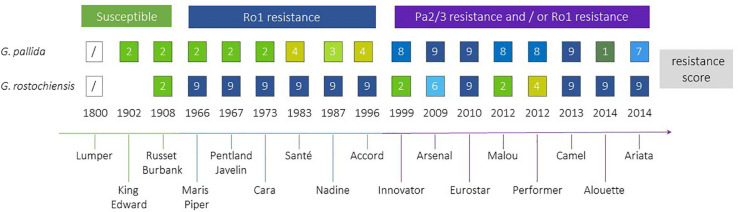
Selected potato cultivars, approximate time when they became commercially available, the introduction of resistances to *Globodera rostochiensis* and *G. pallida*. *H1* resistance is used in all above cultivars against *G. rostochiensis*, whereas for *G. pallida* several sources of resistance have been introgressed. PCN resistance scores from 1 to 9 indicate increasing resistance levels from susceptible (1) to highly resistant (9) (EPPO, 2006).

Efforts to breed resistance to PCN have been ongoing since the middle of the 20th century (Figure 4). The first cross to introgress the *H1* resistance source, which confers resistance to Ro1 and Ro4 pathotypes of *G. rostochiensis*, was between *S. tuberosum* ssp. *andigena* CPC 1673 and the variety Kerr’s Pink in 1952. The resistance was found to be simply inherited, and after backcrossing and selection for commercially important traits as well as resistance, the varieties Maris Piper (England) and Pentland Javelin (Scotland) were released in 1966 and 1967, respectively. Maris Piper remains one of the dominant commercial cultivars in the United Kingdom. The *H1* resistance has been widely deployed in breeding and is the most frequently used source of resistance to *G. rostochiensis* (Przetakiewicz and Milczarek, 2017) in current European and North American potato varieties. This resistance source has proven to be extremely durable to date.

Breeding for resistance to *G. pallida* has been complicated by the more complex nature of most resistance sources to this species and the higher genetic variability of *G. pallida* populations outwith South America, compared to *G. rostochiensis*. A resistance source, denoted *H2*, found in the diploid wild species *S. multidissectum* was shown to confer resistance to some *G. pallida* populations. This resistance was transferred to cultivated potato by interploidy crosses (Bradshaw et al., 1996). However, this gene has not been widely deployed as it does not provide strong resistance to the Pa2/3 populations of *G. pallida* that are the most prevalent in Europe. Breeding efforts in the United Kingdom and mainland Europe focused on the South American diploid species *S. vernei*, which includes accessions with quantitative resistance to both PCN species. Resistance derived from *S. vernei* has been introgressed independently in United Kingdom and mainland European breeding programmes, but it took many years to develop cultivars showing high levels of resistance. Chromosome doubling of *S. vernei* was performed by chemical means. The resulting tetraploid plants were crossed for several generations to cultivars and breeding lines over 30 years, leading to the eventual release of varieties (e.g., Innovator, Arsenal, Eurostar), which contain high levels of resistance to *G. pallida* Pa1, 2/3 populations. The *Grp1* resistance has also been used by breeders in Europe to produce cultivars such as Iledher, Seresta, and Aveka. However, *G. pallida* populations are already able to overcome this resistance (Grenier et al., 2020).

A third source of resistance for *G. pallida* to be successfully targeted in United Kingdom breeding programmes is the so called *H3* source, originating from the landrace *S. tuberosum* spp. *andigena* (CPC 2775 and CPC 2802). It was originally thought to be determined by a single gene, but subsequent analysis has shown that *H3* is genetically controlled by QTLs on chromosomes IV (locus subsequently known as *GpaIV*) and XI (Bryan et al., 2002, 2004). It took over 20 years (but only 2 breeding generations) to produce the partially resistant cultivar Eden and breeding line 12601ab1, which has a higher level of resistance than Eden to pathotypes Pa2/3. The latter also has excellent processing traits, leading to its heavy use in United Kingdom breeding programmes. Several cultivars carrying *GpaIV* have been released with moderate levels of resistance to Pa2/3, such as Vales Everest, Midas, Olympus and Rocket.

Ongoing efforts in potato breeding are aimed at “pyramiding” various sources of PCN resistance into single genotypes. Most of the known sources of resistance to *G. pallida* provide only partial resistance, and moreover are genetically complex, involving the action of more than one gene. The value of pyramiding different loci conferring *G. pallida* resistance has been shown in previous studies, notably that of Caromel et al. (2005) who showed that “stacking” two separate QTLs from the wild species *S. sparsipilum* into individual genotypes resulted in a stronger resistance phenotype than either QTL deployed individually. The ideal scenario would be to combine *H1* with at least two of the major effect QTLs conferring *G. pallida* resistance (e.g., *H2, GpaIV, GpaV*). The successful accomplishment of this goal would be facilitated by the existence of breeding lines with high “dosages” of the relevant functional alleles, as well as genetic markers diagnostic for both the presence and dosage of the functional alleles of these genes.

### Diagnostic Molecular Markers Linked to PCN Resistance and Their Applications

Progress in the genetic analysis of PCN resistance sources led to the mapping of these sources, and in some cases the functional alleles have been isolated (Table 1). This also allowed the development of molecular markers tightly linked to the resistance phenotypes that can be deployed in potato breeding. These markers enable more informed breeding approaches that do not rely solely on time-consuming and progress-limiting phenotypic assessments. The use of MAS in breeding for PCN resistance has been well adopted in most breeding programmes. A brief history of the main resistance mapping efforts and marker development is presented below.

**TABLE 1:** List of described genes and QTLs involved in PCN resistance and their references.

**Species of origin**	**Name**	**Chromosome**	**Species**	**Pathotype**	**References**
**Genes**					
*S. spegazzinii*	*Gro1–4*	VII	*G. rostochiensis*	Ro1	Paal et al., 2004
*S. tuberosum* ssp. *andigena*	*Gpa2*	XII	*G. pallida*	Pa2/3	Rouppe van der Voort et al., 1997; van der Vossen et al., 2000
*S. pimpinellifolium*	*Hero*	IV	*G. rostochiensis*, *G. pallida*	Ro1 Pa2/3	Ernst et al., 2002
**QTLs**					
*S. multidissectum*	*H2*	V	*G. pallida*	Pa1 Pa2/3	Phillips et al., 1994; Strachan et al., 2019
*S. sparsipilum*	*Gpa^*V*^_*spl*_*	V	*G pallida*	Pa2/3	Caromel et al., 2005
	*Gpa^*XI*^_*spl*_*	XI	*G pallida*	Pa2/3	Caromel et al., 2005
*S. spegazzinii*	*Gpa*	V	*G. pallida*	Pa2/3	Kreike et al., 1994; Caromel et al., 2005
	*GpaM1*	V	*G. pallida*	Pa2/3	Caromel et al., 2003
	*GpaM2*	VI	*G. pallida*	Pa2/3	Caromel et al., 2003
	*GpaM3*	XII	*G. pallida*	Pa2/3	Caromel et al., 2003
	*Gro 1.2*	X	*G. rostochiensis*	Ro1	Kreike et al., 1993; Park et al., 2019
	*Gro 1.3*	XI	*G. rostochiensis*	Ro1	Kreike et al., 1993
	*Gro 1.4*	III	*G. rostochiensis*	Ro1	Kreike et al., 1996
*S. tarijense*	*Gpa^*XIl*^_*tar*_*	XI	*G. pallida*	Pa3	Tan et al., 2009
*S. tuberosum* ssp. *andigena*	*H1*	V	*G. rostochiensis*	Ro1 Ro4	Bakker et al., 2004; Finkers-Tomczak et al., 2011
	*H3*	IV	*G pallida*	Pa2/3	Bryan et al. (2004)
*S. vernei*	*GpaV*	V	*G. pallida*	Pa2/3	Rouppe van der Voort et al., 2000; van Eck et al., 2017
	*Gpa VI*	IX	*G. pallida*	Pa2/3	Rouppe van der Voort et al., 2000; Bryan et al., 2002
	*GroVI*	*V*	*G. rostochiensis*	Ro1, Ro4	Jacobs et al., 1996
*S.tuberosum, S. oplocense, S. vernei* (3 different) and *S. tuberosum* ssp. *andigena*	*Grp1*	V	*G. rostochinesis*, *G. pallida*	Ro5 Pa2/3	Rouppe van der Voort et al., 1998; Finkers-Tomczak et al., 2009
*S. tuberosum* ssp. *andigena* and S. *vernei*	*Ro2_A*	V	*G. rostochiensis*	Ro2	Park et al., 2019
	*Ro2_B*	V	*G. rostochiensis*	Ro2	Park et al., 2019
	*Pa2/3_A*	V	*G. pallida*	Pa2/3	Park et al., 2019
	*Pa2/3_B*	X	*G. pallida*	Pa2/3	Park et al., 2019
*S. tuberosum* ssp. *andigena* and *S. vernei*	*Gpa IV*	IV	*G. pallida*	Pa2/3	Bradshaw et al., 1998

The *H1* gene was first localised on chromosome V using restriction fragment length polymorphism (RFLP) mapping technology (Gebhardt et al., 1993). Further attempts to clone *H1* have led to the development of several tightly linked markers (Finkers-Tomczak et al., 2011). The RFLP marker CP113, linked in coupling to *H1*, was deployed to test for the presence of the marker in 53 tetraploid German potato varieties with unknown sources of resistance. This was perhaps the earliest attempt of using molecular markers to survey PCN resistance in potato. Unfortunately, this marker failed to detect the presence of *H1* in resistant varieties, highlighting the need for validating the “transferability” of markers to populations other than those in which the marker was developed. This is a common problem in outbreeding crop species showing low levels of linkage disequilibrium. Two further markers, TG689 and 57R have been reported and deployed for *H1* PCN resistance screening, showing more than 90% congruence between the marker assay and the PCN-resistance phenotype (Biryukova et al., 2008; Milczarek et al., 2011; Schultz et al., 2012). The marker TG689 has been used successfully in a breeding programme which led to the potato cultivar Missaukee, a processing cultivar with resistance to late blight, verticillium wilt and *G. rostochiensis* (Douches et al., 2010). The presence or absence of a 452 bp amplicon for the marker 57R proved to be the most effective diagnostic marker for the prediction of PCN resistance conferred by *H1* (Park et al., 2018). This marker successfully identified *H1* in 90 resistant potato cultivars and in 299 out of 300 from potato breeding programmes, and correctly categorised all susceptible potato cultivars (Schultz et al., 2012). Further development of single nucleotide polymorphisms (SNP) markers diagnostic for *H1* has been reported by Meade et al. (2020) who deployed a highly effective pooled “whole-genome resequencing” approach using previously deployed markers (in this case 57R) to identify novel sequence polymorphisms that could be converted into a more generic competitive allele-specific PCR format than the gel-based system used for 57R.

Attempts to develop diagnostic molecular markers for *GpaIV* and *GpaV* major QTLs have been only partially successful, progress being hampered by the quantitative nature of these resistances. A PCR-based marker, SPUD 1636, was reported for the *GpaV* QTL which was developed by converting a closely linked amplified fragment length polymorphism (AFLP) marker (Bryan et al., 2002). This presence/absence marker generates a 226 bp amplicon from resistant sources, and the diagnostic value of the SPUD1636 marker to identify sources of resistance was demonstrated for some accessions of *S. vernei* as well as varieties and breeding lines (Bryan et al., 2002). The marker has also been adapted for MAS in a Spanish resistance breeding programme (Ortega and Lopez-Vizcon, 2012) and used for resistance screening by Sudha et al. (2016). However, it has turned out to be inefficient in selecting breeding lines (Milczarek et al., 2011). Subsequently, Sattarzadeh et al. (2006) reported the development of the PCR marker “HC” to tag the same resistance QTL from *S. vernei*. Rather than developing markers based on single polymorphic sites linked to the QTL, the authors performed “haplotype” analysis on a set of seven closely linked SNPs, all located at the QTL region spanning over several hundred kilobases (kb) and each showed significant associations with resistance phenotypes. Of the three haplotypes identified, haplotype “c” stood out as the one associated in coupling with the resistance. Two SNPs that were specific to the haplotype “c” were targeted to develop the PCR marker HC. Tests of the HC marker on 56 potato varieties beyond the experimental population indicated that HC was highly diagnostic for the presence of the *S. vernei*-derived resistance to *G. pallida* pathotype Pa2/3. The HC marker, since its development, has been used widely to screen for resistance (Sharma et al., 2014; Bhardwaj et al., 2019; Sudha et al., 2019). It generally shows good agreement with phenotypic tests although discrepancies occasionally occur. Schwarzfischer et al. (2010) showed that HC is an appropriate diagnostic tool to identify resistance in diverse German potato varieties. It has also been discovered that many European breeding lines contain the HC marker, indicating that this marker has been widely used in resistance breeding (Sattarzadeh et al., 2006).

For the “*H3*” source, the first marker to be used in breeding was the microsatellite STM3016, for which a particular allele (STM3016-122/177) was diagnostic for the *GpaIV* gene on chromosome IV (Moloney et al., 2010). Further work, based on the targeted resequencing of bacterial-artificial chromosome (BAC) clones from the region, led to the development of the PCR marker contig237, as well as three others (Moloney et al., 2010), which were highly predictive for *GpaIV* in a breeding population.

Other resistance sources to PCN targeted for marker development, are the gene *Gro1–4*, for which a sequence characterised amplified region (SCAR) marker was developed following efforts to isolate the gene (Paal et al., 2004). Additional primer pairs were developed (Asano et al., 2012) but neither has been reliable for screening purposes. A further source of resistance to *G. rostochiensis* is the gene *GroVI*, identified in *S. vernei* (Jacobs et al., 1996) and mapped to chromosome V at the same region as *H1*. Its allelic relation to *H1* remains unclear. Two reported SCAR markers were developed by converting two RAPD markers, U14 and X02 that flank *GroVI*, which generate amplicons of 366 and 854 bp, respectively. The two markers mapped 19 cM distal and 4 cM proximal to *GroVI* (Jacobs et al., 1996). Milczarek et al. (2011) used the two markers to screen a collection of 72 cultivars for resistances and generated the expected bands but found them to be undiagnostic as they were observed in both resistant and susceptible cultivars. Like *Gro1–4*, the *GroVI* resistance has not been deployed as widely as *H1*, probably due to overlapping with *H1* in its resistance specificity to the most common pathotype Ro1.

**FIGURE 5:**
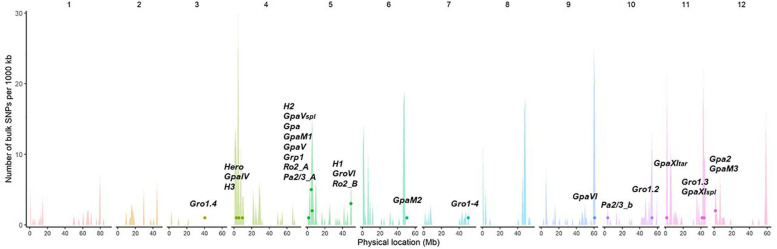
Potato cyst nematodes (PCN) resistance loci align with known R gene clusters. The unequal distribution and position of predicted NB-LRR genes in the genome of DM is illustrated in the histogram, with spikes indicating clusters of resistance. The approximate relative location in the reference genome DM (PGSC v4.03) of known nematode resistance genes and QTLs are displayed as dots. The histogram spikes indicate numbers of predicted NB-LRR genes per 1,000 kb across the potato genome.

The majority of described resistance genes or QTLs for PCN are located within resistance “gene clusters” within the potato genome. Almost half of the described QTLs from different potato wild species map to the same region on chromosome V, where resistances for other potato pathogens are also described. This region, defined by RFLP markers GP21 and GP179 is called a “hotspot” for resistances (Gebhardt and Valkonen, 2001). Therefore, some of these resistances might share a common origin and possibly developed in ancestors and later evolved by diversification in wild potato species (Finkers-Tomczak et al., 2009). The resistances *Grp1* and *GpaV* have been mapped to the same chromosomal region, raising the possibility that they might be conferred by the same genetic factors (Finkers-Tomczak et al., 2009). Figure 5 illustrates these findings, showing the alignment and clustering of known nematode *R*-gene loci to higher numbers of predicted NB-LRRs, or hotspots of resistance, in the doubled monoploid *S. tuberosum* group *Phureja* clone DM 1-3 516 R44 (DM), which was developed as the first potato reference genome.

### Potato Genomics Tools Applied to the Analysis of PCN Resistance

The first nematode *R* gene, *Hs1^*pro*–1^*, was identified in sugar beet against the beet cyst nematode, *Heterodera schachtii*, in 1997 and relied on analysing chromosomal breakage points alongside a “satellite” marker (Cai et al., 1997). However, subsequent genetic analysis showed that resistance does not co-segregate with the presence of this gene (Sandal et al., 1997). Other plant resistances were studied with the aid of bulked segregant analysis (BSA) using molecular markers such as RFLPs; cleaved amplified polymorphic sequences (CAPS) and AFLPs (Michelmore et al., 1991). In 1998, the tomato gene *Mi-1* was cloned, which provides resistance to both root knot nematodes (*Meloidogyne incognita*, *M. javanica*, *M. arenaria*) and several phloem-feeding insects, through the use of yeast-artificial chromosome libraries (Vos et al., 1998). Similarly, *Gpa2* from potato was mapped to a 115kb region linked to a sequence-related NB-LRR gene, which provides resistance to potato virus X (Rouppe van der Voort et al., 2000; van der Vossen et al., 2000). *Gro1–4* was isolated in a similar way (Paal et al., 2004). The identification of the *Hero* locus from tomato, although initially defined with RFLP markers in a segregating population, was more complex than *Gpa2* or *Gro1–4* (Ganal et al., 1995). A total of 14 potential candidate NB-LRRs were identified by sequencing 19 cosmids spanning the 180kb locus. Based on cosmid sequence information, these gene candidates were narrowed down by analysis of open reading frames, and the use of specific PCR markers. This allowed the functional analysis of just three potential candidate genes to identify the *Hero* gene conferring broad spectrum resistance to *G. rostochiensis* and some *G. pallida* pathotypes (Ernst et al., 2002). For other resistance genes, a map-based BSA approach has proven to be unsuitable. The *H1* gene for example, mapped to the distal end of chromosome V that contains numerous NB-LRR homologues, has very limited recombination and a lack of co-linearity between different haplotypes (Bakker et al., 2004; Finkers-Tomczak et al., 2011). While this gene has not yet been isolated, despite great efforts to do so, newer approaches and technologies may allow this and other genes to be isolated from such complex loci.

The three PCN resistance genes cloned so far belong to the NB-LRR family of *R-*genes. *Gro1–4* encodes an NB-LRR protein with a Toll-interleukin receptor domain at the N-terminus (Paal et al., 2004), while *Hero* (Ernst et al., 2002) and *Gpa2* (van der Vossen et al., 2000) encode CC-NB-LRR proteins, which have a coiled-coil domain at the N-terminus. *Gro1–4* and *Gpa2* originate from *S. spegazzinii* and *S. tuberosum* ssp. *andigena*, respectively, while the *Hero* resistance gene is derived from the wild tomato species *S. pimpinellifolium*. None of the other important PCN resistances have yet been isolated, despite significant efforts to do so. Nevertheless, mapping work has led to the development of several genetic markers for resistance that are used in potato breeding programmes, as described above.

With the availability of a reference potato genome (The Potato Genome Sequencing Consortium, 2011) followed by rapid advancements and cost reductions in next-generation sequencing (NGS), modern genotyping methods, for example genotyping-by-sequencing (GBS) (Elshire et al., 2011) with novel genetic approaches, such as genome-wide association studies (GWAS) (Visscher et al., 2012) for QTL discovery and marker-trait analyses, have emerged and have been applied to potato (e.g., Sharma et al., 2018). These approaches provide much higher genetic resolution than the previously used “low density” approaches. They also allow for high throughput analyses and larger populations to be rapidly screened (Deschamps et al., 2012). The release of the potato reference genome aimed to provide a resource for identifying important genes (Sharma et al., 2013) including NB-LRRs. Although this reference has been invaluable in genetic studies, it only offers an incomplete picture, particularly in highly repetitive regions or in the case of NB-LRR genes where the reference is from a largely susceptible background. Thus, more recently, potato genomes have been sequenced from the homozygous diploid inbred potato clone M6 of *S. chacoense*, which is used as a breeding line and in genetics analysis (Jansky et al., 2014). A heterozygous clone, RH, has also solved haplotype blocks with third generation sequencing technology (Leisner et al., 2018; Zhou et al., 2020). These additional genomes will allow a greater resolution and understanding of NB-LRR loci and aid in the isolation and cloning of target genes.

An alternative to conventional “genome-wide” mapping approaches that overcomes some of the limitations of the potato reference genomes is provided by targeted enrichment sequencing. This process reduces the genome complexity to the most likely candidates with the help of specific “baits” designed to a subset of all potato genes. For potato, the following enrichment strategies have been adopted to date and comprise whole exome capture (WEC), generic-mapping enrichment sequencing (GenSeq) which targets single copy, conserved genes dispersed throughout the potato genome, two defence gene related capture approaches, resistance gene enrichment sequencing (RenSeq), and receptor-like protein enrichment sequencing (Jupe et al., 2013; Giolai et al., 2016; Chen et al., 2018; Lin et al., 2020). RenSeq was developed to allow high resolution SNP markers to be obtained for the detection and mapping of NB-LRRs (Jupe et al., 2013). RenSeq has also proven important to identify full length candidate NB-LRRs in combination with long-read sequencing (Witek et al., 2016). Furthermore, when used as a diagnostic tool (dRenSeq), the application informs resistance breeders, providing a full and detailed picture of the known NB-LRRs present in wild species and parental material (Van Weymers et al., 2016; Armstrong et al., 2019).

The most recent approach to isolate and map resistance genes is independent of a reference genome and takes advantage of *K-*mers in conjunction with association studies. *K*-mers are subreads of a length = *K* and, unlike SNPs, can inform on small haplotypes. The genetic mapping processes of *K*-mers are similar to GWAS by analysing diverse accessions for association between sequence polymorphisms and traits, but this can be conducted independently of a reference genome. *K*-mer studies based on RenSeq derived reads are referred to as AgRenSeq and have been used, for example, to isolate four resistance genes in wheat (Arora et al., 2019). Other, non-RenSeq-based *K*-mer approaches have also recently been used to map resistance against wart disease in potatoes (Prodhomme et al., 2019).

As sequencing methods continue to improve, so will the outputs for the *de novo* assembly of genomes or loci of interest, particularly in highly repetitive regions and for the many potato species with high levels of heterozygosity. Indeed, newly emerging and rapidly improving long-read sequencing technologies such as Oxford Nanopore and PacBio, are already starting to provide potential solutions for potato genomics and new potato genomes are being released (van Lieshout et al., 2020; Zhou et al., 2020). With these technological advances and the computational tools that facilitate ever more complex analyses, mapping and cloning of resistance genes should become more routine in the future.

## Discussion

While host resistance deployment remains a preferred strategy to control pests and pathogens of potato, introgression of resistance genes from wild and landrace species into commercially successful cultivars remains a considerable challenge. On the one hand, adaptation of pests and pathogens to overcome resistance can negate all of the efforts of breeders. On the other hand, meeting commercial requirements in which resistance is only one of many characters the breeder must deliver, can also negate their efforts. Education, incentives and interventions may all be required in order for PCN resistant cultivars to be more widely grown by the potato industry and for consumer acceptance. More options in cultivars are urgently needed that meet different requirements of the potato markets. Rapid advances in genomics improving transferability of markers for specific traits, such as PCN resistance, but also for a wide range of other agronomically important characters, should revolutionise potato breeding in the future.

One reason for the relative lack of success in breeding for *G. pallida* resistance is that efforts have tended to focus on the larger QTL and neglect the smaller effect loci (chromosomes IX and XI for *S. vernei* and *H3*, respectively). Caromel et al. (2005) demonstrated the potential of MAS for introgression of *G. pallida* resistance genes derived from *S. sparsipilum* in a potato breeding programme by “stacking” two separate QTLs into individual genotypes. This resulted in a more-robust resistance phenotype than was obtained by the presence of a single QTL. This study and those of Dalton et al. (2013) and Rigney et al. (2017) underpin the basis for current efforts to “pyramid” resistance genes and QTLs into the same genetic background. Combined with the implementation of genomic selection (GS), which takes advantage of the availability of genome-wide high density markers to estimate the breeding value and the analysis of more complex traits, molecular markers are gaining significant traction in the breeding of potatoes (Slater et al., 2016).

Resistance genes that act to protect potatoes from PCN are a precious commodity. There are limited numbers of resistance genes that are amenable to introgression that will likely make a significant impact in the foreseeable future for the management of *G. pallida*. While there is an urgent need for new cultivars with *G. pallida* resistance that meet commercial requirements, more effort will be needed to ensure that resistance remains effective and durable.

## Author Contributions

UG and VB coordinated the manuscript. All authors listed have made direct intellectual contributions to the article and its underpinning research and have approved it for publication.

## Conflict of Interest

The authors declare that the research was conducted in the absence of any commercial or financial relationships that could be construed as a potential conflict of interest.

## Footnotes

1 http://www.nemadecide.com

2 https://pcncalculator.ahdb.org.uk

3 https://www.aphis.usda.gov/aphis/ourfocus/planthealth/plant-pest-and-disease-programs/pests-and-diseases/nematode/pcn/pcn-home
